# Case Report: Aortic Regurgitation of Postocclusion and Long-Term Outcome Following PDA Correction in an Adult Dog

**DOI:** 10.3389/fvets.2022.848313

**Published:** 2022-03-14

**Authors:** Woong-Bin Ro, Hee-Myung Park, Doo-Won Song, Heyong-Seok Kim, Ga-Won Lee, Jin-Ho Kang, Chan-Ho Jo, Min-Hee Kang

**Affiliations:** ^1^Department of Veterinary Internal Medicine, College of Veterinary Medicine, Konkuk University, Seoul, South Korea; ^2^Royal Dog and Cat Medical Center, Seoul, South Korea; ^3^Department of Bio-Animal Care, Jangan University, Hwaseong, South Korea

**Keywords:** canine, patent ductus arteriosus, ACDO, cardiac intervention, postocclusion aortic insufficiency, complication

## Abstract

A 9-year-old intact female Maltese dog was admitted for further evaluation of previously diagnosed patent ductus arteriosus (PDA). The dog showed severe coughing and exercise intolerance. On physical examination, a grade VI/VI continuous heart murmur was auscultated. Thoracic radiography demonstrated cardiomegaly, pulmonary overcirculation, and moderate bronchointerstitial pattern. Echocardiography revealed severe dilation of the left ventricle and atrium, decreased left ventricular contractility, and left-to-right PDA. On electrocardiography (ECG), R amplitude was increased. Computed tomographic angiography revealed type IIA PDA. The serum N-terminal pro-B-type natriuretic peptide (NT-proBNP) concentration was >10,000 pmol/L. Transarterial occlusion was performed and the Amplatz® Canine Duct Occluder was successfully deployed. On echocardiography 48 h after the procedure, aortic regurgitation (AR) and residual ductal flow were noted. Long-term follow-up on clinical signs, physical examination, radiography, echocardiography, ECG, and serum NT-proBNP were evaluated until 30 months after correction of PDA. The clinical indices of physical examination, thoracic radiography, echocardiography, ECG, and serum NT-proBNP concentration were improved, although the postocclusion AR and residual ductal flow persisted. The dog followed up without clinical signs for 41 months following the correction. To our knowledge, this is the first case report to demonstrate quite a long time of follow-up (41 months) in an older dog with transarterial occlusion of PDA with postocclusion AR and residual flow.

## Introduction

Patent ductus arteriosus (PDA) is one of the most common congenital cardiac disease in dogs, in which persistent fetal ductal structure between the aorta and main pulmonary artery (PA) results in left-sided cardiac volume overload, congestive heart failure, and eventually death (1, 2). Currently, percutaneous transcatheter occlusion of PDA using Amplatz^®^ Canine Duct Occluder (ACDO) is one of the most widely used treatment methods in dogs (3, 4). The advantages of ACDO procedure include high complete occlusion rate, easy deployment, minimal invasiveness, and low complication rate (4, 5). However, complications such as residual PDA shunt, device embolization, device migration, and endocarditis have been reported after ACDO procedure in dogs (4, 6–8).

In humans, the occurrence of aortic regurgitation (AR) after percutaneous PDA occlusion using coil or Amplatz^®^ Duct Occluder has been reported in some patients (9, 10). Identification of this postocclusion AR is important for managing patients and determining their prognosis, and it is necessary to distinguish it from ARs due to other causes such as infective endocarditis or connective tissue diseases (9).

However, postocclusion AR has been rarely reported in dogs after percutaneous closure of PDA, without detailed description and long-term follow-up results (11, 12). Therefore, the clinical relevance and long-term outcome of this postocclusion AR remain unknown in dogs. The present report describes clinical indices of long-term follow-up of AR following transarterial PDA occlusion using ACDO in an older dog, including physical examination, blood work, radiography, echocardiography, electrocardiography (ECG), and cardiac biomarker.

## Case Description

A 9-year-old intact female 2.41-kg Maltese dog was admitted for evaluation of previously diagnosed PDA. The dog showed severe coughing and exercise intolerance despite prior treatment with pimobendan [0.25 mg/kg, orally (PO) every 12 h], ramipril (0.125 mg/kg, PO every 24 h), furosemide (1 mg/kg, PO every 12 h), sildenafil (1 mg/kg, PO every 12 h), and codeine (0.3 mg/kg, PO every 12 h).

On physical examination, continuous heart murmur (grade VI/VI) was auscultated at the left heart base area. The dog showed intermittent panting after coughing, and resting respiratory rate was 24 breaths/min in the absence of coughing. Body condition score was 2/5, heart rate was 166 beats/min, and blood pressure was within normal range (systolic pressure 111 mm Hg, diastolic pressure 67 mm Hg; Cardell 9402 Veterinary Monitor, Midmark, Tampa, FL, USA). Complete blood count and serum chemistry revealed mild normocytic normochromic anemia, mild hypoalbuminemia, mildly increased blood urea nitrogen, and aspartate aminotransferase (AST), mild hypocalcemia, hypertriglyceridemia, increased lactate dehydrogenase (LDH), and mild hyperkalemia (Supplementary Table 1).

**Figure 1:**
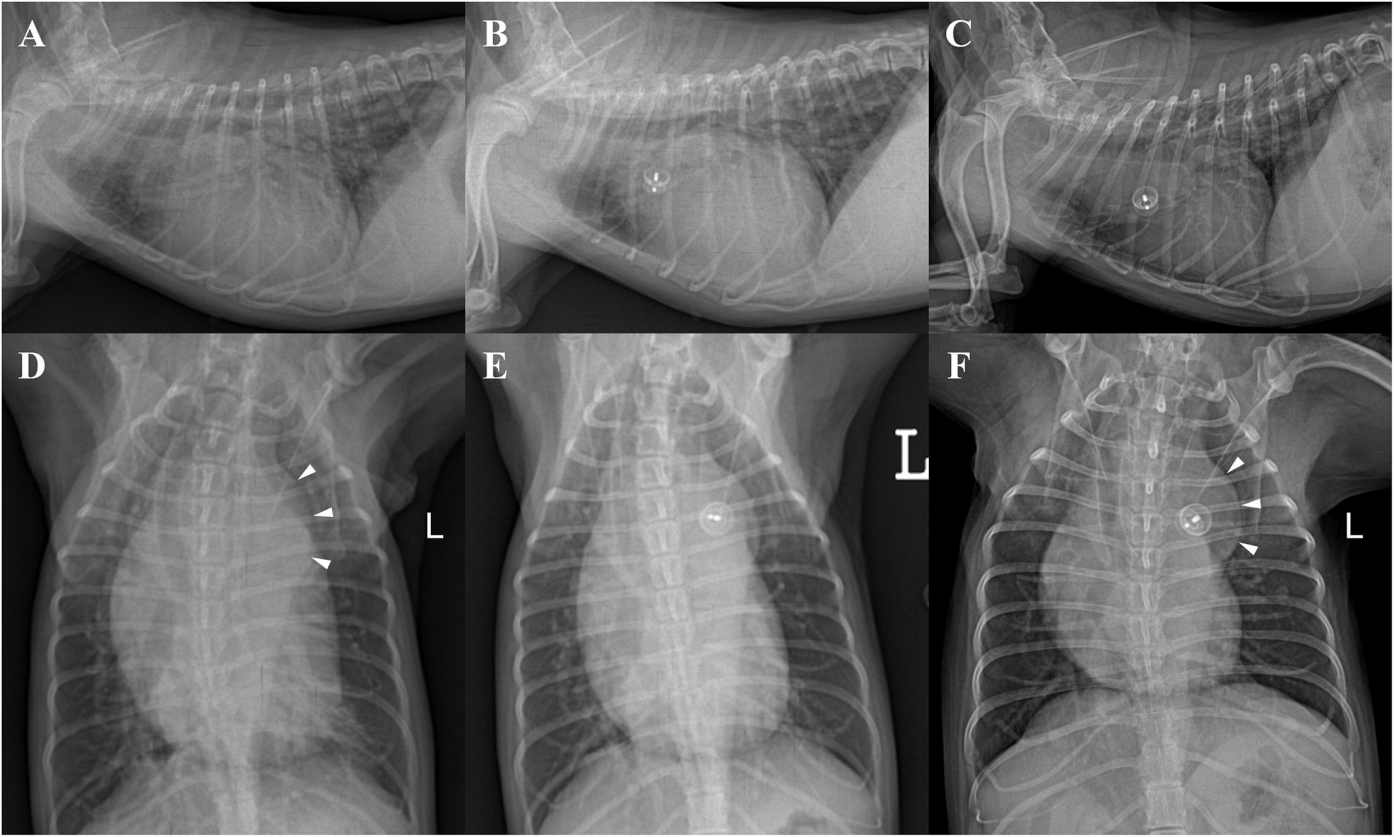
Thoracic radiographs of a 9-year-old 2.41-kg Maltese dog with patent ductus arteriosus in right lateral **(A–C)** and ventrodorsal **(D–F)** views. **(A,D)** Preprocedural radiographs showing severe enlargement of all four cardiac chambers (VHS = 13.4), leftward bulge of descending aorta (aneurysmal bulge, arrowheads), pulmonary overcirculation, tracheal deviation to the dorsal and right side, and moderate bronchointerstitial pattern on perihilar and caudal lung region. **(B,E)** Radiographs obtained 48 h after ACDO deployment showing decreased cardiomegaly (VHS = 12.3) and pulmonary overcirculation. **(C,F)** Radiographs obtained 30 months after ACDO deployment. Note the significant decrease in cardiac size (VHS = 11), pulmonary overcirculation, and interstitial lung pattern compared with the preprocedural radiographs and similarly maintained aneurysmal bulge (arrowheads). ACDO, Amplatz^®^ Canine Duct Occluder; VHS, vertebral heart score.

Thoracic radiography demonstrated severe enlargement of all four cardiac chambers, a vertebral heart score (VHS) of 13.4, leftward bulge of descending aorta (aneurysmal bulge), increased pulmonary circulation, tracheal deviation to the dorsal and right side, and moderate bronchointerstitial pattern on perihilar and caudal lung lobes (Figures 1A,D).

**Figure 2:**
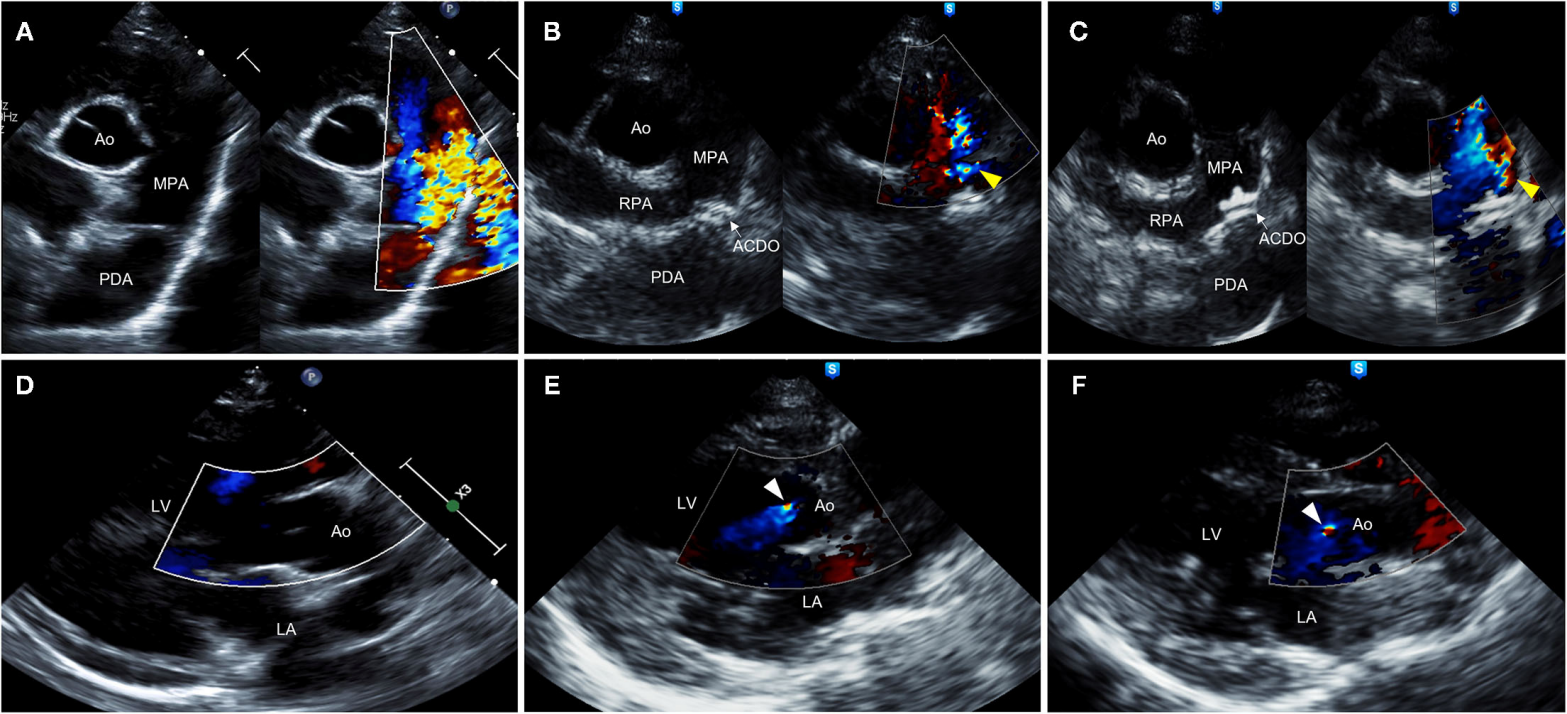
Right parasternal short-axis **(A**–**C)** and long-axis **(D**–**F)** view B-mode and color-flow Doppler images on transthoracic echocardiography of a 9-year-old 2.41-kg Maltese dog with type IIA PDA. **(A,D)** Preprocedural echocardiographic images showing turbulent flow from PDA into MPA **(A)** and absence of AR **(D)**. **(B,E)** Echocardiographic images obtained 48 h after ACDO deployment showing appropriately positioned ACDO, mild residual flow (yellow arrowhead) **(B)**, and AR (white arrowhead) **(E)**. **(C,F)** Echocardiographic images obtained 30 months after ACDO deployment showing unchanged ACDO position, persistent mild residual flow (yellow arrowhead) **(C)**, and persistent AR (white arrowhead) **(F)**. ACDO, Amplatz^®^ Canine Duct Occluder; Ao, aorta; AR, aortic regurgitation; LA, left atrium; LV, left ventricle; MPA, main pulmonary artery; PDA, patent ductus arteriosus; RPA, right pulmonary artery.

Transthoracic echocardiography (TTE) (EPIQ 7 Ultrasound System; Philips Medical Systems, Andover, MA, USA) revealed severe dilation of left ventricle (LV) and left atrium (LA) [ratio of left atrial to aortic root diameter (LA/Ao) = 2.21] and decreased LV contractility [fractional shortening (FS) = 15.7%]. Mild mitral valve thickening and mitral regurgitation (MR) (peak velocity = 3.93 m/s) were observed in color and continuous wave Doppler. PA diameter was significantly enlarged (1.40 cm) compared with the aortic root (Ao) diameter of 1.04 cm. A turbulent flow coming from PDA toward main PA (left-to-right shunt) was observed (peak velocity = 5.49 m/s), with minimal ductal diameter (MDD) of 5.15 mm (Figure 2). Peak aortic flow velocity was increased (2.21 m/s), and diastolic dysfunction stage 2 (pseudonormal diastole) was diagnosed based on the transmitral flow and mitral annular motion profiles in diastole (13). Pulmonary-to-systemic flow ratio (Qp/Qs) was significantly increased to 2.11. Specific echocardiographic values are shown in Table 1. On ECG, sinus rhythm with heart rate of 166 beats/min and increased R amplitude (2.8 mV; reference range, ≤ 2.5 mV) was observed (Supplementary Table 2). Computed tomographic angiography and three-dimensional volume-rendering visualized type IIA PDA coursing from ventral margin of descending aorta to dorsal margin of main PA (Supplementary Figure 1) (14). The serum N-terminal pro–B-type natriuretic peptide (NT-proBNP) concentration was >10,000 pmol/L (reference value, 0–900 pmol/L) (IDEXX Laboratories Inc., Westbrook, ME, USA).

**Table 1:** Echocardiographic results before, 48 h, and 30 months after ACDO deployment in a 9-year-old dog with patent ductus arteriosus.

**Variables**	**Preprocedure**	**Post 48 h**	**Post 30** **months**	**Reference** **range**
FS (%)	15.70	11.70	36.70	25–50
EF (%)	33.90	26.10	68.50	59–83
EDVI	318.37	238.50	147.07	44–117
ESVI	210.41	176.26	46.38	9–38
Ao (cm)	1.04	1.37	1.19	–
PA (cm)	1.40	1.33	1.26	–
LA/Ao	2.21	1.55	1.46	0.8–1.3
LVIDDN	2.85	2.53	2.08	1.35–1.73
LVIDSN	2.36	2.19	1.29	0.79–1.14
LVWDN	0.38	0.37	0.38	0.33–0.53
LVWSN	0.59	0.63	0.63	0.53–0.78
IVSTDN	0.45	0.37	0.37	0.33–0.52
IVSTSN	0.57	0.51	0.66	0.48–0.71
E/A	1.37	NM	0.83	0.98–1.70
IVRT (ms)	60	NM	70	41–65
E′/A′	0.89	NM	0.95	–
E/E′	18.72	NM	6.37	<12
Diastolic dysfunction	Stage 2	NA	Stage 1	–
Aortic flow (m/s)	2.21	NM	0.67	–
MR flow (m/s)	3.93	4.82	1.66	–
AR flow (m/s)	No AR	Detected	2.41	–
Ductal/residual flow (m/s)	5.49	Detected	0.97	–
Qp/Qs	2.11	NA	0.81	–

*ACDO, Amplatz^®^ Canine Duct Occluder; Ao, aortic root diameter; AR, aortic regurgitation; Detected, detected on color Doppler but velocity not measured; E/A, ratio of early to late diastolic transmitral peak velocity; E′/A′, ratio of early to late diastolic mitral annular motion peak velocity; E/E′, ratio of early diastolic transmitral peak velocity to early diastolic mitral annular motion peak velocity; EDVI, end-diastolic volume index; EF, ejection fraction; ESVI, end-systolic volume index; FS, fractional shortening; IVRT, isovolumic relaxation time; IVSTDN, normalized value of interventricular septal thickness in diastole; IVSTSN, normalized value of interventricular septal thickness in systole; LA/Ao, ratio of left atrial to aortic root diameter; LVIDDN, normalized value of left ventricular end-diastolic diameter; LVIDSN, normalized value of left ventricular end-systolic diameter; LVWDN, normalized value of left ventricular free wall thickness in diastole; LVWSN, normalized value of left ventricular free wall thickness in systole; MR, mitral regurgitation; NA, not applicable; NM, not measured; PA, pulmonary artery diameter; Qp/Qs, pulmonary-to-systemic flow ratio*.

The interventional procedure was performed as previously reported (15). Angiography was conducted, and the left-to-right shunt was confirmed with MDD of 5 mm and ampulla diameter of 9 mm. For transarterial occlusion of the PDA, the right femoral artery was exposed *via* surgical cut-down, and 4F vascular long introducer (Check-Flo Performer^®^ Introducer Sets; Cook Medical Inc., Bloomington, IN, USA) and 4-mm ACDO (Infiniti Medical, Menlo Park, CA, USA) were used because of small size of the dog's femoral artery. The device was successfully deployed, and the continuous murmur disappeared, although grade III systolic murmur remained. The dog recovered from anesthesia without any event and was discharged the following day.

The dog was reexamined 48 h after the procedure. The dog owner reported that the clinical signs including coughing and exercise intolerance were improved. On thoracic radiography, decreased cardiomegaly (VHS = 12.3) and improved pulmonary overcirculation were observed (Figures 1B,E). The TTE showed appropriately positioned ACDO and mild residual flow from the PDA (Figure 2B). Compared with the preprocedural TTE, LV internal dimensions, LV volume indices, LA dimension, PA diameter, and LV contractility were decreased, whereas the Ao diameter and MR flow velocity were increased (Table 1). In addition, AR was confirmed in color Doppler, which was not previously observed. After the reexamination, previously prescribed medications were discontinued except for pimobendan (0.25 mg/kg, PO every 12 h).

After 30 months from the ACDO deployment, the dog was presented for recheck. The dog had no clinical signs and returned to normal. On physical examination, a grade II to-and-fro murmur was auscultated at the point of maximum intensity of left heart base. The body weight was increased to 2.7 kg (BCS 3/5), heart rate was 114 beats/min, resting respiratory rate was 24 breaths/min, and blood pressure was within normal range (systolic pressure 114 mm Hg, diastolic pressure 82 mm Hg). Complete blood count and serum chemistry revealed mild normocytic hypochromic anemia, mild hyperproteinemia, hypoalbuminemia, hyperglobulinemia, mildly increased AST, moderately increased creatine kinase and LDH, and mild hyperkalemia (Supplementary Table 1).

Thoracic radiography revealed significant decrease in cardiac size (VHS = 11), pulmonary overcirculation, and interstitial lung pattern compared with the preprocedural radiographs and similarly maintained aneurysmal bulge (Figures 1C,F).

**Figure 3:**
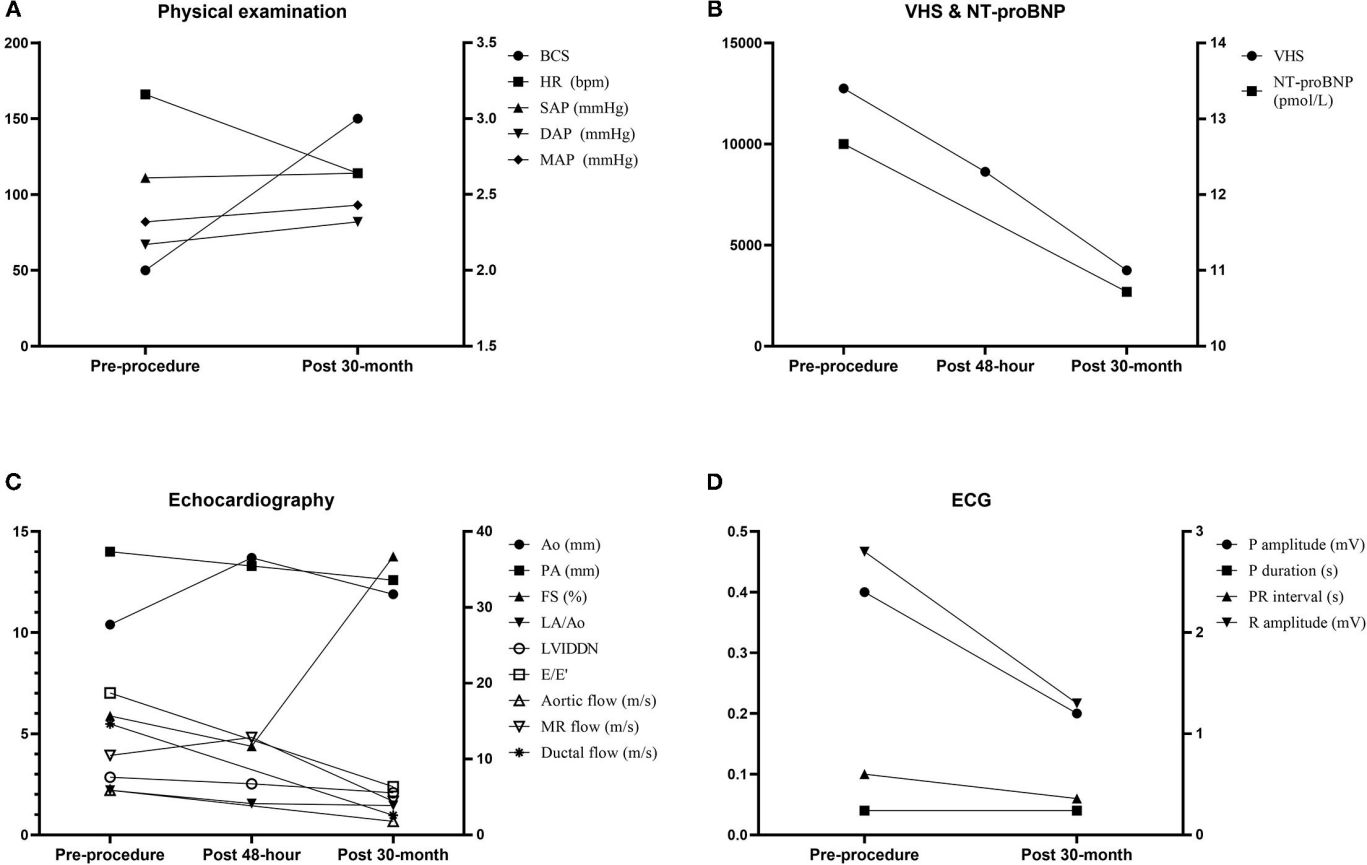
Changes in the long-term follow-up results including physical examination **(A)**, VHS and cardiac biomarker NT-proBNP **(B)**, echocardiography **(C)**, and electrocardiography **(D)**. Ao, aorta; BCS, body condition score; DAP, diastolic arterial pressure; E/E′, ratio of early diastolic transmitral peak velocity to early diastolic mitral annular motion peak velocity; FS, fractional shortening; HR, heart rate; LA/Ao, ratio of left atrial to aortic root diameter; LVIDDN, normalized value of left ventricular end-diastolic diameter; MAP, mean arterial pressure; MR, mitral regurgitation; NT-proBNP, N-terminal pro–B-type natriuretic peptide; PA, pulmonary artery; SAP, systolic arterial pressure; VHS, vertebral heart score.

On the TTE, LV internal dimensions, LV volume indices, LA dimension, and PA diameter were further decreased, and LV contractility was markedly increased compared with the preprocedural and post−48-h TTE results (Table 1). The Ao diameter was decreased compared with the post−48-h result, although still larger than preprocedural diameter. Peak aortic flow velocity, Qp/Qs, and LV filling pressure index (E/E′, ratio of early diastolic transmitral peak velocity to early diastolic mitral annular motion peak velocity) were decreased to normal range, and diastolic dysfunction was improved to stage 1 (delayed relaxation) (13). The MR flow velocity was significantly decreased compared with previous examinations. The AR and residual flow from PDA observed in the post−48-h TTE were still present. On ECG, the R amplitude was decreased to normal range, and other indices including P amplitude and PR interval were also decreased within normal range compared with the preprocedural results (Supplementary Table 2). The serum NT-proBNP concentration was decreased to 2,689 pmol/L. The dog has remained healthy without clinical signs until the time of this writing, which is 41 months after the procedure. Changes in the long-term follow-up results including physical examination, radiography, echocardiography, ECG, and cardiac biomarker are shown in Figure 3.

## Discussion

Generally, in dogs, PDA has poor prognosis with a high mortality rate within 1 year if untreated, but long-term survival is possible if corrected at an early age (1, 16). In case of dogs that presented at older age, PDA correction is frequently avoided because of the risk of anesthesia, although PDA occlusion and subsequent clinical improvement have been previously reported in older dogs as well (17, 18). However, long-term outcomes of more than 1 year after ACDO procedure have not been reported in older dogs. The present case described favorable long-term outcome after ACDO procedure in an old dog, in which the improved condition of the dog was confirmed through various clinical indices. Therefore, this case may suggest that even if the PDA occlusion was not performed at an early age, it is necessary to be corrected even in older dogs. However, further studies on a larger number of cases are required to evaluate this.

In humans, AR is reported to be one of the possible complications following PDA occlusion (9, 10, 19, 20). Although the exact incidence remains unknown, postocclusion AR is known to occur rarely in human patients with PDA (20). However, in a study in PDA patients with MDD >1.5 mm (9), postocclusion AR occurred in approximately one-quarter of the patients, which suggests an association between the development of AR and the size of the MDD. In humans, AR following PDA occlusion is usually trivial to mild with benign prognosis, which is often resolved within 1–3 years (9). However, severe AR that is hemodynamically relevant and AR lasting more than 6 years have also been reported in humans (9, 20). Therefore, identifying postocclusion AR is considered clinically important in humans, and distinguishing it from other diseases with poor prognosis, such as infective endocarditis, rheumatic fever, or connective tissue disease, is also necessary (9, 10, 20).

AR after Amplatz^®^ Duct Occluder deployment was more likely to develop in human patients with hemodynamically significant left-to-right PDA shunts, and the MDD indexed to body weight was significantly larger in patients with newly developed AR compared with patients with no AR (9). In another human study (10), larger PDA shunt size corrected for body weight and higher Qp/Qs were significant risk factors for development of AR following coil occlusion of PDA. Based on the MDD and the Qp/Qs, the dog in the present case had a large PDA shunt size and hemodynamically significant left-to-right shunt (13, 21). These factors are considered to have contributed to the development of postocclusion AR in this case, similar to the prior human studies (9, 10).

In this case, the older age of the dog may have been a contributing factor to the persistence of postocclusion AR. As mentioned earlier, most of the postocclusion AR in humans is known to resolve spontaneously within 3 years (9, 20). However, in this case, the postocclusion AR persisted for more than 41 months. As the diagnosis and subsequent treatment of PDA are delayed, most older dogs with PDA present with advanced cardiac remodeling and deterioration of cardiac function (17, 18), and these cardiac deformation and dysfunction are more likely to be persistent after PDA closure in older dogs (1). Therefore, it is speculated that the postocclusion AR in the dog of the present case may be persistent.

According to previous studies (19, 22), a sudden rise of systemic arterial blood pressure by an acute elevation of systemic vascular resistance and increased vascular capacitance of proximal aorta were suggested as the mechanism of postocclusion AR. In addition, deformation of the aortic root structure by sudden hemodynamic change after PDA closure was also proposed as a cause of persistent AR (9). In the dog in this case, aortic root diameter was significantly enlarged after ACDO procedure, indicating an increase in intra-aortic pressure and structural deformation. After 30 months from the procedure, the aortic root diameter was decreased but remained larger than the preprocedural diameter, and this persistent structural deformation is considered to be one of the possible causes of persistent AR in this case. In addition, the increase in diastolic arterial blood pressure observed 30 months after ACDO procedure would have also contributed to the development and persistence of AR.

The dog in the present case showed residual ductal flow after the ACDO procedure, which persisted more than 30 months. In the dog in this case, an ACDO smaller than the PDA ductal size (80% of MDD) was used because of the small size of the dog's femoral artery, which is thought to have contributed to the occurrence of persistent residual ductal flow. In addition, the older age of the dog in this report also may have affected the development of residual flow. In human patients with PDA (23), age-related changes in ductal tissue and morphology that can cause postocclusion complications were reported. Also, older age was suggested as a contributing factor in a dog with delayed embolization of ACDO (24). In this case, the prolonged PDA in this case could have caused changes in the ductal tissue and structure, leading to development of residual ductal flow.

In this case, reduction of FS was observed 48 h after the procedure, whereas significant improvement of FS was confirmed after 30 months. This reduction of systolic function after PDA occlusion was previously reported in dogs and is known to be a result of increased afterload and decreased LV preload (25). This also means that AR and residual ductal flow were still present, even though the afterload was decreased 30 months after the procedure, which supports the irreversible deformation of the aortic and ductal structures. The increase and decrease in MR can be explained by the same mechanism as FS, and it can be speculated that the unmeasured velocities of AR and residual ductal flow on 48-h postprocedure would have been faster than those on 30-month postprocedure.

As the PDA correction was not made until late age, the dog in this case showed severe deterioration of cardiac function and remodeling. In addition, because of the concurrence of AR, residual ductal flow, and MR, volume overload may have persisted even after the PDA occlusion. This persistence of volume overload and consequent cardiac damage could be confirmed by increased serum NT-proBNP concentration 30 months after the procedure, despite marked improvement compared with preprocedure. Nevertheless, the dog in this case did not show any clinical signs, and most of the clinical indices including physical examination, radiography, echocardiography, and ECG could be fairly maintained with only pimobendan administration for a long period of 30 months. This outcome represents relatively benign nature of postocclusion AR, which should be distinguished from AR caused by infective endocarditis that may also occur as a postocclusion complication (9).

To the authors' knowledge, this is the first case report describing the long-term follow-up outcome of an older dog with persistent AR and residual ductal flow after ACDO procedure. In older PDA dogs with large and hemodynamically relevant shunts, it should be considered that postocclusion AR may occur after ACDO procedure. Identifying postocclusion AR in PDA dogs would be helpful in prognosis evaluation and patient monitoring, especially when combined with other conditions such as residual ductal flow or MR. Despite several postocclusion complications, ACDO procedure could improve the quality of life and extend the survival time in an elderly PDA dog.

## Data Availability Statement

The original contributions presented in the study are included in the article/Supplementary Material, further inquiries can be directed to the corresponding author.

## Ethics Statement

Ethical review and approval was not required for the animal study because this case describes clinical management of an individual animal. Written informed consent was obtained from the owners for the participation of their animals in this study.

## Author Contributions

W-BR, J-HK, and C-HJ contributed to case management. W-BR, H-MP, D-WS, and M-HK contributed to case analysis. W-BR, H-SK, and G-WL contributed to data curation. W-BR contributed to manuscript preparation. W-BR, H-MP, and M-HK contributed to manuscript editing. All authors contributed to the article and approved the submitted version.

## Conflict of Interest

The authors declare that the research was conducted in the absence of any commercial or financial relationships that could be construed as a potential conflict of interest.

## Publisher's Note

All claims expressed in this article are solely those of the authors and do not necessarily represent those of their affiliated organizations, or those of the publisher, the editors and the reviewers. Any product that may be evaluated in this article, or claim that may be made by its manufacturer, is not guaranteed or endorsed by the publisher.
