# Supplementary material for: Case Report: Aortic Regurgitation of Postocclusion and Long-Term Outcome Following PDA Correction in an Adult Dog
Source: Front Vet Sci. 2022 Mar 14;9:848313. doi: 10.3389/fvets.2022.848313 (PMC8963992; doi:10.3389/fvets.2022.848313)
Supplement: Supplementary file 1 [file Data_Sheet_1.docx]

**Supplementary Materials**

**Supplementary Table 1.**Blood examination results before and 30 months after ACDO deployment in a 9-year-old dog with patent ductus arteriosus

| **Variables** | | **Pre-procedure** | **Post 30 months** | **Reference range** |
| --- | --- | --- | --- | --- |
| **Complete blood count** | |  |  |  |
|  | WBC (10x3/L) | 8.7 | 7.4 | 6-17 |
|  | RBC (10x6/L) | 5.2 | 4.4 | 5.5-8.5 |
|  | Hemoglobin (g/dL) | 12 | 9 | 12-18 |
|  | Hematocrit (%) | 35 | 31 | 37-55 |
|  | MCV (fL) | 68 | 71 | 60-77 |
|  | MCH (pg) | 22.9 | 21.5 | 19.5-24.5 |
|  | MCHC (g/dL) | 34 | 31 | 32-36 |
|  | Platelet (10x3/L) | 383 | 447 | 200-500 |
| **Serum chemistry** | |  |  |  |
|  | Total protein (g/dL) | 6.0 | 7.3 | 4.9-7.2 |
|  | Albumin (g/dL) | 2.0 | 1.9 | 2.3-3.9 |
|  | Globulin (g/dL) | 4.0 | 5.3 | 2.5-4.5 |
|  | Glucose (mg/dL) | 87 | 88 | 67-147 |
|  | BUN (mg/dL) | 39 | 26 | 5-30 |
|  | Creatinine (mg/dL) | 0.9 | 0.9 | 0.5-1.5 |
|  | ALP (U/L) | 44 | 147 | 20-155 |
|  | ALT (U/L) | 14 | 16 | 3-50 |
|  | AST (U/L) | 44 | 59 | 10-37 |
|  | GGT (U/L) | 5 | 7 | 4-25 |
|  | Total bilirubin (mg/dL) | 0.1 | 0.1 | 0.1-0.7 |
|  | Calcium (mg/dL) | 8.9 | 9.1 | 9.1-11.7 |
|  | Phosphorus (mg/dL) | 4.3 | 3.6 | 2.4-6.4 |
|  | Triglyceride (mg/dL) | 130 | 89 | 21-116 |
|  | Cholesterol (mg/dL) | 278 | 189 | 127-340 |
|  | CK (U/L) | 138 | 248 | 25-167 |
|  | LDH (U/L) | 533 | 766 | 65-269 |
|  | Lipase (U/L) | 35 | 75 | 5-90 |
|  | Na (mmol/L) | 142 | 148 | 139-149 |
|  | K (mmol/L) | 5.3 | 5.3 | 3.5-5.2 |
|  | Cl (mmol/L) | 111 | 113 | 103-117 |

ACDO, Amplatz® Canine Duct Occluder; ALP, alkaline phosphatase; ALT, alanine aminotransferase; AST, aspartate aminotransferase; BUN, blood urea nitrogen; CK, creatine kinase; Cl, chloride; GGT, gamma glutamyl transferase; K, potassium; LDH, lactate dehydrogenase; MCH, mean corpuscular hemoglobin; MCHC, mean corpuscular hemoglobin concentration; MCV, mean corpuscular volume; Na, sodium; RBC, red blood cell; WBC, white blood cell.

**Supplementary Table 2.**Electrocardiographic results before and 30 months after ACDO deployment in a 9-year-old dog with patent ductus arteriosus

| **Variables (lead II)** | **Pre-procedure** | **Post 30-month** | **Reference range** |
| --- | --- | --- | --- |
| P duration (s) | 0.04 | 0.04 | ≤ 0.04 |
| P amplitude (mV) | 0.40 | 0.20 | ≤ 0.40 |
| PR interval (s) | 0.10 | 0.06 | 0.06 - 0.13 |
| QRS duration (s) | 0.04 | 0.04 | ≤ 0.05 |
| R amplitude (mV) | 2.80 | 1.30 | ≤ 2.50 |
| ST segment (mV) | - | - | ≤ 0.20 |
| QT interval (s) | 0.16 | 0.16 | 0.15 – 0.25 |
| T wave (mV) | 0.4 | 0.3 | ≤ 1/4 R amplitude |

ACDO, Amplatz® Canine Duct Occluder.

**Figure legend**

**Supplementary Figure 1.**Pre-procedural computed tomography (CT) images showing type IIA patent ductus arteriosus (arrows) in a 9-year-old 2.41-Kg Maltese dog. **(A)** A three-dimensional volume rendering CT image. **(B)** A sagittal CT angiography. **(C)** A dorsal CT angiography. **(D)** A transverse CT angiography. Ao, aorta; PA, pulmonary artery.
