# Supplementary figures and images for: Case Report: Aortic Regurgitation of Postocclusion and Long-Term Outcome Following PDA Correction in an Adult Dog
Source: Front Vet Sci. 2022 Mar 14;9:848313. doi: 10.3389/fvets.2022.848313 (PMC8963992; doi:10.3389/fvets.2022.848313)

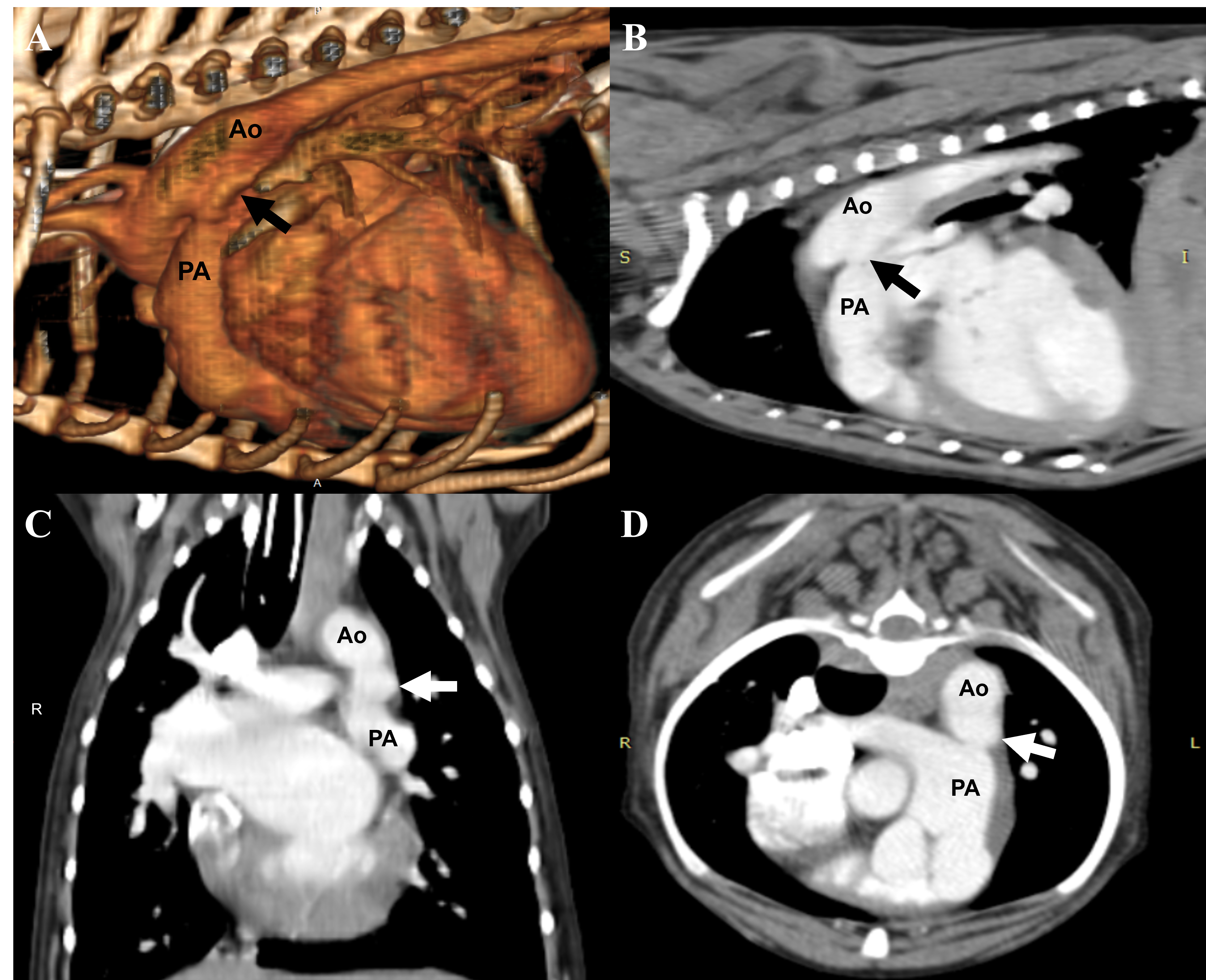

Supplement: Supplementary file 2 [file Image_1.TIF]
